# Experimental Study of Electroosmosis in Rock Cores Based on the Dual Pressure Sensor Method

**DOI:** 10.3390/s24092832

**Published:** 2024-04-29

**Authors:** Chenggang Yin, Wei Guan, Hengshan Hu

**Affiliations:** 1 College of Intelligent Manufacture, Taizhou University, Taizhou 317000, China; 2 Department of Astronautics and Mechanics, Harbin Institute of Technology, Harbin 150001, China; guanw@hit.edu.cn (W.G.); hhs@hit.edu.cn (H.H.)

**Keywords:** core sample, electroosmotic experiment, pressure sensor, electroosmotic pressure coefficient

## Abstract

Electroosmotic experiments obtain the electroosmotic pressure coefficient of a rock sample by measuring the excitation voltage at both ends of the sample and the pressure difference caused by the excitation voltage. The electroosmotic pressure is very weak and buried in the background noise, which is the most difficult signal to measure in the dynamic-electric coupling experiment, so it is necessary to improve its signal-to-noise ratio. In this paper, for the low signal-to-noise ratio of electroosmotic pressure, the dual pressure sensor method is proposed, i.e., two pressure sensors of the same type are used to measure electroosmotic pressure. Two different data extraction methods, Fast Fourier Transform and Locked Amplification, are utilized to compare the dual pressure sensor method of this paper with the existing single pressure sensor method. The relationship between the electroosmotic pressure coefficient and the excitation frequency, mineralization, permeability, and porosity is analyzed and discussed.

## 1. Introduction

The pore surfaces of fluid-saturated pore media selectively absorb some ions of the fluid electrolyte, leaving a net surplus of mobile ions in the pore, thus constituting a double electric layer. Under a pressure difference, the net surplus ions generate an electrical signal with fluid motion, a phenomenon known as the flow potential effect. On the contrary, under the action of an electric field, the net surplus ions hold the nearby solution hostage to flow, a phenomenon known as the electroosmotic effect. These two coupling effects are collectively referred to as the electrokinetic effect [1,2,3]. These two kinds of electrokinetic effects have important applications in the directions of seismic early warning, seismic exploration and electrokinetic logging in the field of geophysics. Among them, the electroosmotic experiment obtains the electroosmotic pressure coefficient of a core sample by measuring the excitation voltage at both ends of the sample and the pressure difference caused by the excitation voltage. The electroosmotic pressure is very weak and buried in the background noise, which is the most difficult signal to measure in the dynamic-electric coupling experiment. Carrying out electroosmotic experiments in pore media in the laboratory, measuring the electroosmotic pressure coefficients of rock samples, and analyzing their relationship with the parameters of rock samples are the basis for the application of the above kinetic-electric effect in the field of geophysics. Therefore, it is of great interest to carry out electroosmotic experiments on rock samples.

Thompson and Gist [4] were the first to measure electroacoustic exploration signals at shallow depths at the Friendswood Experimental Range in the USA. They used a 20 kW power amplifier to transmit conventional electrical pulse signals near the surface and then measured the electrical pulse-induced acoustic signals in a well 300 m below ground level. Touchard et al. [5] applied a direct current of 500 V for 4 h to the ends of rock samples saturated with a 0.3 mol/L lithium chloride solution, and then calculated the amount of lithium ions that flowed through the rock samples by measuring the change in the concentration of the lithium chloride solution on both sides of the samples before and after the voltage was applied. The amount of lithium ions flowing through the rock sample was used to assess the magnitude of the seepage from the electro-osmosis. Hornbostel and Thompson [6] encoded the electric pulse source for electroacoustic exploration in both linear and nonlinear sequences to improve the signal-to-noise ratio of the electric pulse induced acoustic signals. Thompson et al. [7] used a 350 kW power waveform synthesizer to transmit the encoded electric pulse signals near the ground surface and then measured the signal-to-noise ratio in a well 1000 m below the ground surface. The signal-to-noise ratio was then improved by using a 350 kW power waveform synthesizer to transmit the coded electrical pulse signals. Chen et al. [8] analyzed the loose permeable strata in the frozen section of the shaft and compared and analyzed them with on-site hydrological data. Hu et al. [9] were the first to simulate electroacoustic logging and calculate the electroacoustic wave field in homogeneous formations. Electroacoustic logging is slightly different from electroacoustic exploration at shallow depths: the former transmits electrical pulse signals in wells, which can be measured in deeper formations; the latter transmits electrical pulse signals near the surface, which can emit stronger electrical pulses; and both measure electrical pulse-induced acoustic signals induced by electroosmotic effects in well bores. Guan and Hu [10] simulated electroacoustic wavefields for horizontally stratified formations in a horizontally stratified model with electric dipole excitation by using a time-domain finite difference method. The electroacoustic logging wavefields in horizontally layered formations were simulated using the time-domain finite-difference method. Zhu et al. [11] measured electroacoustic logging in a horizontally layered model in the laboratory and measured electrical pulse-induced acoustic signals, which showed that the measured induced acoustic field was a Stoney wave. Zyserman et al. [12] used finite elements to simulate the wavefields in electroacoustic logging of methane hydrate formations and showed that electroacoustic logging is sensitive to the concentration of methane hydrate. Bruell et al. [13] studied the use of the electroosmotic effect to aid in oil extraction in the laboratory. Bruell et al. studied the use of electroosmotic effects to assist oil extraction in the laboratory and showed that hydrocarbons with higher water solubility are easily transported and vice versa. Ghazanfari et al. [14] simulated the use of electroosmotic effects to assist oil extraction in oil-water two-phase formations. 

In summary, the bilayer kinetic effect is receiving increasing attention from the geophysical community and its applications are becoming more widespread. Measuring the electroosmotic pressure coefficient of rock samples in the laboratory is the basis for the application of the kinetic effect. Therefore, this paper proposes the dual pressure sensor method for the low signal-to-noise ratio of the electroosmotic pressure, i.e., the electroosmotic pressure coefficient is obtained by measuring the electroosmotic pressure using two pressure sensors of the same type; secondly, it compares with the existing single-pressure-sensor method; and lastly, it analyzes and discusses the relationship between the electroosmotic pressure coefficient and each parameter.

## 2. Electroosmotic Theory

**Figure 1:**
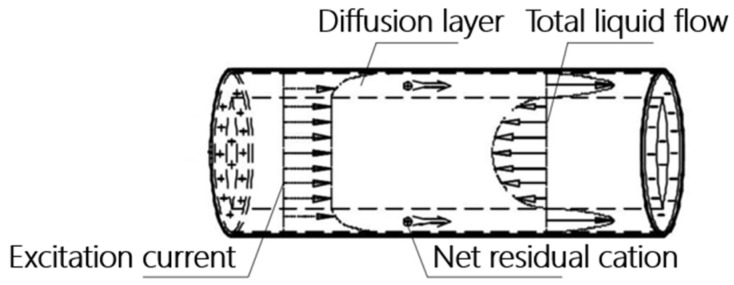
The schematic of the electroosmotic effect.

The net excess cations within the pores of a fluid-saturated pore medium produce an acoustic field under the action of an electric field, a phenomenon known as the electroosmotic effect. As shown in Figure 1, the diffusion layer facilitates the movement of the net surplus cations under the influence of the excitation current, carrying the nearby solution and moving in the direction of the electric field lines, thus generating a macroscopic liquid flow, known as electroosmotic seepage. When the parameters of the fluid saturated pore medium are kept constant, the electroosmotic percolation density is proportional to the field strength of the excitation voltage, i.e.,

(1) $$\frac{v_{E}}{\phi A}=L_{21}\frac{\Delta U_{E}}{l},$$

where $\frac{v_{E}}{\left(\phi A\right)}$ is the electroosmotic seepage density, $L_{21}$ is the electroosmotic effect of the kinetic coupling coefficient, $\Delta U_{E}$ is the excitation voltage, that is, applied to the pore medium at both ends of the potential difference. The two ends of the pore medium are sealed, then the fluid will accumulate at one end of the pore medium under the transportation of the electroosmotic flow, and then form a pressure difference opposite to the direction of the electroosmotic flow, which is called the electroosmotic pressure $\Delta P_{E}$. The electroosmotic pressure obeys Darcy’s law [15]. For electroosmotic experiments on rock samples, Darcy’s law can be expressed as.

(2) $$\frac{v_{D}}{\phi A}=\frac{\kappa \Delta P_{E}}{\eta l},$$

where $v_{D}$ is the seepage rate, κ is the permeability of the pore medium, and η is the viscosity coefficient of the solution within the pores.

The negative of the ratio of the electroosmotic pressure to the excitation voltage at a single frequency is denoted as “electroosmotic pressure coefficient at frequency” $C_{E}(f_{0})$, i.e.,

(3) $$C_{E}(f_{0})=-{\left.\frac{\Delta P_{E}}{\Delta U_{E}}\right|}_{f=f_{0}},$$

The negative sign in the above equation indicates that the electroosmotic pressure and the excitation voltage are in opposite directions. When the excitation frequency $f_{0}$ is small enough, the system reaches equilibrium, when the total seepage in the orifice is zero, i.e.,

(4) $$v_{E}=v_{D},$$

The negative of the ratio of electroosmotic pressure and excitation voltage at the equilibrium of the system is denoted as “electroosmotic pressure coefficient” $C_{E0}$, i.e.,

(5) $$C_{E0}={\left.-\frac{\Delta P_{E}}{\Delta U_{E}}\right|}_{v_{E}=v_{D}},$$

From the conditions of Equation (4), the system can definitely reach equilibrium when the excitation frequency tends to zero, i.e.,

(6) $$\underset{f\to 0}{lim}C_{E}(f)=C_{E0},$$

Therefore, $C_{E0}$ is the low-frequency limit of $C_{E}(f_{0})$.

According to Equations (1), (2), (5) and (6),

(7) $$L_{21}=\frac{\kappa }{\eta }C_{E0},$$

## 3. Dual Pressure Sensor Method

The electroosmotic pressure signal is very weak and buried in a very strong background noise. Obtaining a high signal-to-noise ratio of electroosmotic pressure is the difficulty and focus of electroosmotic experiments. Theoretically, there are two ways to improve the signal-to-noise ratio: one is to increase the amplitude of the electroosmotic pressure; the other is to suppress the background noise. The amplitude of electroosmotic pressure is limited by the amplitude of the excitation voltage, which cannot be arbitrarily increased. From Equation (5), theoretically, an increase in the excitation voltage can be equal to the amplitude of the electroosmotic pressure in the equilibrium state. However, for excitation frequencies on the order of millihertz, an excitation voltage of a few volts is sufficient to cause electrolysis of the solution and release of gases, while electrolysis also alters the pH and conductivity of the solution [15]. The gases produced by electrolysis, as well as changes in the pH and conductivity of the solution change the value of the electroosmotic pressure coefficient. For experiments on rock samples saturated with highly mineralized solutions, excessive excitation currents (>5 mA/cm^2^) can lead to a significant increase in the temperature of the rock samples within a safe excitation voltage range [16]. It can be seen that both excitation voltage and excitation current have upper limits. In this paper, the excitation voltage is less than or equal to 0.2 V and the excitation current is less than or equal to 1 mA/cm^2^. In this case, increasing the electroosmotic pressure by increasing the excitation voltage is not favorable to the accuracy of the experimental data, i.e., it is not feasible to increase the magnitude of the electroosmotic pressure.

Other scholars have used the single pressure sensor method to measure the electroosmotic pressure, i.e., a single pressure sensor is used to convert the electroosmotic pressure from a pressure signal to an electrical signal, and then the value of the electroosmotic pressure is extracted by a lock-in amplifier [15]. The lock-in amplifier has two input signals, one is the measured signal and the other is the reference signal. The lock-in amplifier first multiplies the two input signals by a phase-sensitive detector, and then the output signal of the phase-sensitive detector is filtered with a low-pass filter to obtain the output signal of the low-pass filter, where ‘amplitude’ and ‘phase’ represent the amplitude and phase of the measured signal, respectively. The amplitude and phase of the measured signal can be obtained by using two sets of phase-sensitive detectors and a low-pass filter with a 90° difference in the reference signals, which is referred to as a quadrature vector-type lock-in amplifier (hereinafter referred to as a lock-in amplifier) [17,18].

**Figure 2:**
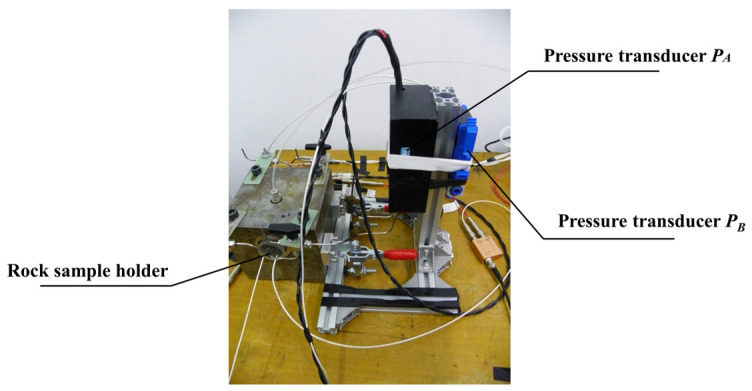
Two pressure sensors were used in the electroosmotic experiment.

In order to suppress the background noise of electroosmotic pressure, the dual pressure sensor method is proposed in this paper, as shown in Figure 2. The dual pressure sensor method uses two pressure sensors to measure the electroosmotic pressure and the background noise separately, and then the data acquisition card is used for analog/digital conversion, and finally, the electroosmotic pressure and its error estimation are obtained by weighted difference, spline fitting and fast Fourier transform. The lab measures at room temperature. The lateral side of the sample is sealed by rubber under about 1.2 MPa in a rock sample holder, and two ends of the sample link to two input ports of the pressure transducer $P_{A}$, respectively.

### 3.1. Theory of Dual Pressure Sensor Method

In this paper, two low range pressure sensors of the same type are used to measure electroosmotic pressure. The pressure sensors are used to measure electroosmotic pressure signals with background noise,

(8) $$\Delta P_{A}=\Delta P_{E}+\Delta P_{A\_noise},$$

where $\Delta P_{A}$ is the measured value of the pressure sensor $P_{A}$ and $\Delta P_{A\_noise}$ is the background noise of the pressure sensor $P_{A}$. The pressure sensor $P_{B}$ is used to synchronize the monitoring of the background noise, the

(9) $$\Delta P_{B}=\Delta P_{B\_noise},$$

where $\Delta P_{B}$ is the measured value of the pressure sensor $P_{B}$ and $\Delta P_{A\_noise}$ is the background noise of the pressure sensor $P_{B}$.

In this paper, it is found experimentally that there is a strong correlation between the background noise of two pressure sensors although they are not numerically equal. In order to estimate the background noise $\Delta P_{A\_noise}$ by measuring the background noise $\Delta P_{A\_noise}$, it is necessary to obtain the conversion coefficients $C_{pt}$ of the two pressure sensors,

(10) $$C_{pt}=\frac{\Delta P_{A\_noise}}{\Delta P_{B\_noise}},$$

From Equation (1) the electroosmotic seepage is zero when there is no excitation voltage at both ends of the rock sample, i.e.,

(11) $${v_{E}|}_{\Delta U_{E}=0}=0,$$

From the principle of electroosmotic effect, the electroosmotic pressure is caused by electroosmotic seepage, i.e.,

(12) $${{\Delta p}_{A}|}_{\Delta U_{E}=0}=0,$$

Substituting Equations (11) and (12) into Equation (8) gives

(13) $${{\Delta p}_{A}|}_{\Delta U_{E}=0}=\Delta P_{A\_noise},$$

Substituting Equations (9) and (13) into Equation (10) gives

(14) $$C_{pt}={\left.-\frac{\Delta P_{E}}{\Delta U_{E}}\right|}_{{\Delta U}_{E}=0},$$

Therefore, the conversion coefficient $C_{pt}$ of the two pressure sensors can be obtained by measuring the pressure signals of the two pressure sensors when there is no excitation voltage.

For the traditional single pressure sensor method, the pressure sensor’s measurement is the electroosmotic pressure signal, i.e.,

(15) $$\Delta P_{E\_SPTM}=\Delta P_{A},$$

where $\Delta P_{E\_SPTM}$ is the electroosmotic pressure signal obtained by the single pressure sensor method.

For the dual pressure sensor method, from Equations (8)–(10),

(16) $$\Delta P_{E\_DPTM}=\Delta P_{A}-C_{pt}\Delta P_{B},$$

where $\Delta P_{E\_SPTM}$ is the electroosmotic pressure signal obtained by the dual pressure sensor method and $C_{pt}\Delta P_{B}$ is the background noise of the pressure sensor $P_{A}$. In this way, the electroosmotic pressure signal can be obtained after suppressing the background noise. 

### 3.2. Determination of Conversion Coefficients for Two Pressure Sensors

**Figure 3:**
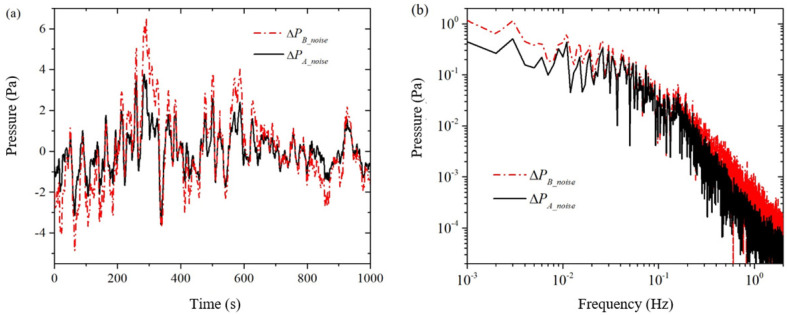
Measurements of the pressure sensor $P_{A}$ and $P_{B}$ when there is no excitation voltage across the core sample. (**a**) time domain; (**b**) frequency domain.

The solid and dashed lines in Figure 3 represent the measured background noise $P_{A}$ and $P_{B}$ for pressure sensors $\Delta P_{A\_noise}$ and $\Delta P_{B\_noise}$, respectively, in a single simultaneous acquisition. As seen from the time-domain signals in Figure 3a, the waveforms of the two pressure sensors are very similar, but the amplitude of the pressure sensor $P_{B}$ is slightly larger than that of pressure sensor $P_{A}$ in terms of amplitude. We made several measurements and found that the time-domain signals of both pressure sensors have nearly identical waveforms but different amplitudes. This shows a good correlation between the background noise of the two pressure sensors. From the frequency domain signal in Figure 3b, it can be seen that the conversion coefficient $C_{pt}$ of the background noise of the two pressure sensors is not a constant: in the middle of the frequency band from 0.01 Hz to 0.2 Hz, the difference in the background noise is relatively small, and from the formula, it can be seen that the conversion coefficient a is larger at this time; at the two ends of the frequency bands from 0.001 Hz to 0.01 Hz and from 0.2 Hz to 2 Hz, the difference of the background noise is relatively larger, at this time the conversion coefficient $C_{pt}$ a is smaller.

**Figure 4:**
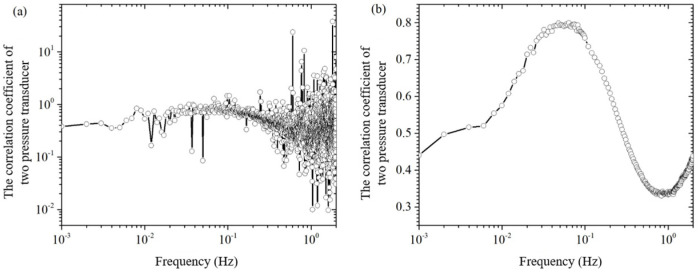
The correlation coefficient of background noise of the pressure sensor $P_{A}$ and $P_{B}$ at different frequencies. (**a**) a point-to-point correlation coefficient; (**b**) an average correlation coefficient in several measurements.

By dividing the amplitudes at the same frequency of the two pressure sensors in Figure 3b, the theoretical conversion coefficient of the two pressure sensors can be obtained, which is called the point-to-point pressure sensor conversion coefficient, denoted as $C_{pt\_p2p}$. As shown in Figure 4a, the amplitude curve of the point-to-point pressure sensor conversion factor oscillates more and more as the frequency increases. This “burr”-like oscillation is caused by the two frequency domain signals in Figure 3b, the peaks and valleys of the wave division (or wave valley and peak division). It is clear that the error in the conversion factor of the point-to-point pressure sensor is too large to be used to estimate the background noise $\Delta P_{B\_noise}$ of the pressure sensor $P_{B}$ from the known background noise $\Delta P_{A\_noise}$ of the pressure sensor $P_{A}$.

In order to eliminate the “burr” on the conversion coefficient curve in Figure 4a, this paper adopts the following methods: (1) As shown in Figure 3, measure the background noise of the two pressure sensors and the two pressure sensors when there is no excitation pressure, and obtain the frequency-domain signals; (2) In the frequency-domain signals, average the two curves with their surrounding points, so that frequency domain curves become relatively smooth; (3) Do point-to-point division on the two averaged smooth frequency domain curves to obtain the averaged conversion coefficients of a single measurement; (4) Repeat steps 1 to 3 to obtain the averaged conversion coefficients of multiple single measurements; and (5) Do averaging on the averaged conversion coefficients of multiple single measurements to obtain the averaged conversion coefficients of multiple measurements, as shown in Figure 4b. From comparison of Figure 4a,b, it can be seen that, through the averaging process, the conversion coefficient curve on the “burr” has been eliminated.

### 3.3. The Measured Signal of the Dual Pressure Sensor Method

**Figure 5:**
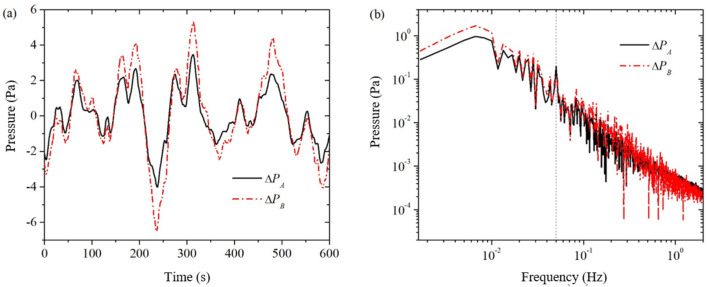
The measured signals of the pressure sensor $P_{A}$ and $P_{B}$ when the excitation voltage is applied across the sample. (**a**) time domain; (**b**) frequency domain.

Figure 5 shows a set of measured signals of the electroosmotic pressure, where the rock sample is sandstone S12 (as shown in Table 1), the mineralization is 0.4 mol/L, the excitation frequency is 0.05 Hz, and the number of excitation cycles is 30 cycles. For this example, the excitation frequency is 0.05 Hz, then the frequency of the electroosmotic pressure is also 0.05 Hz. This is shown as a sine wave with a period of 20 s in the time domain plot and as a peak at 0.05 Hz in the frequency domain (indicated by the vertical line in the figure). As shown in Figure 5a, there are some peaks and valleys in the pressure signal measured by the pressure sensor $P_{A}$ containing electroosmotic pressure $\Delta P_{E}$ as well as strong background noise $\Delta P_{A\_noise}$ (solid line), but these peaks and valleys are not in the period of 20 s, instead, they are similar to the pure background noise measured by the pressure sensor $P_{B}$ (dashed line) in terms of waveforms, but there are some differences in amplitude. This indicates that the electroosmotic pressure signal $\Delta P_{E}$ is essentially drowned in the strong background noise. As shown in Figure 5b, the measured signal ${\Delta P}_{A}$ of the pressure sensor $P_{A}$ (solid line) has a less pronounced peak at the excitation frequency of 0.05 Hz, and is very similar to the pure background noise ${\Delta P}_{B}$ measured by the pressure sensor $P_{B}$ (dashed line) at the rest of the frequency.

### 3.4. Data Processing of Dual Pressure Sensor Method

**Figure 6:**
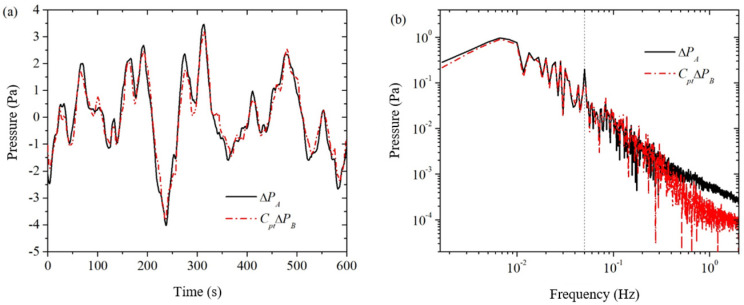
Comparisons between the measured signal with electroosmotic pressure $\Delta P_{A}$ and the corrected background noise $C_{pt}\Delta P_{B}$. (**a**) time domain; (**b**) frequency domain.

Multiplying the obtained averaged conversion factor $C_{pt}$ of the two pressure sensors with the background noise $\Delta P_{B}$ measured by the pressure sensor $P_{B}$ in the frequency domain yields the corrected background noise $C_{pt}\Delta P_{B}$, as shown by the dashed line in Figure 6. In Figure 6a, the corrected background noise $C_{pt}\Delta P_{B}$ is very close to the actual measured signal $\Delta P_{A}$ from the pressure sensor $P_{A}$. This indicates on the one hand that the electroosmotic pressure signal $\Delta P_{E}$ is very weak relative to the background noise, and on the other hand the corrected background noise $C_{pt}\Delta P_{B}$ can relatively well simulate the actual background noise $\Delta P_{A}$ of the pressure sensor $P_{A}$. In Figure 6b, the corrected background noise $C_{pt}\Delta P_{B}$ is very close $\Delta P_{A}$ in the frequency band of 0.3 Hz and below, but the difference between the two is larger in the frequency band above 0.3 Hz. This indicates that the dual pressure sensor method is more applicable to the lower frequency band of 0.3 Hz and below (hereafter referred to as the lower frequency band). Although the dual pressure sensor method for measuring electroosmotic pressure has a range of applicability in the frequency domain, this does not affect its application to the measurement of electroosmotic pressure coefficients. This is because the higher frequency band above 0.3 Hz (hereinafter referred to as the higher frequency band) of the electroosmotic pressure of the background noise of the magnitude of 1 mPa and below, if the higher frequency band of the electroosmotic pressure of the magnitude of 10 mPa and below, then the electroosmotic pressure coefficient is mainly dependent on the lower band of the electroosmotic pressure and the excitation voltage of the ratio of the electroosmotic pressure that is, at this time, the higher band of electroosmotic pressure and not the role of the actual.; If the higher frequency band electroosmotic pressure is off the order of 100 mPa and above, then the higher frequency band electroosmotic pressure itself has enough signal-to-noise ratio, and there is no need for the dual pressure sensor method. In addition, this paper uses the spline fitting interpolation method to correct the drift of the higher frequency band.

**Figure 7:**
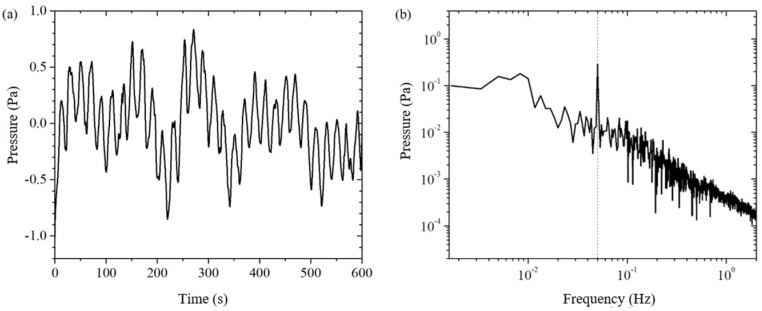
The electroosmotic pressure after weighted difference of the measured signals of two pressure sensors. (**a**) time domain; (**b**) frequency domain.

Subtracting the measured signal $\Delta P_{A}$ from the pressure sensor $P_{A}$ with the corrected background noise $C_{pt}\Delta P_{B}$ from the pressure sensor $P_{B}$ gives the electroosmotic pressure signal after using the weighted difference, noted as $\Delta P_{E\_DPTM}^{\prime }$. In Figure 7a, we can clearly see the electroosmotic pressure signal with a period of 20 s. It can be seen that the signal-to-noise ratio of the electroosmotic pressure is improved by the weighted difference of the dual pressure sensors. In Figure 7b, the electroosmotic pressure signal at 0.05 Hz is clearly much stronger than the residual background noise around it. By reading the amplitude of the excitation frequency signal and the critical frequency noise in the frequency domain plot, it can be obtained $\Delta P_{E\_DPTM}^{\prime }=0.288\pm 0.027\;Pa$.

**Figure 8:**
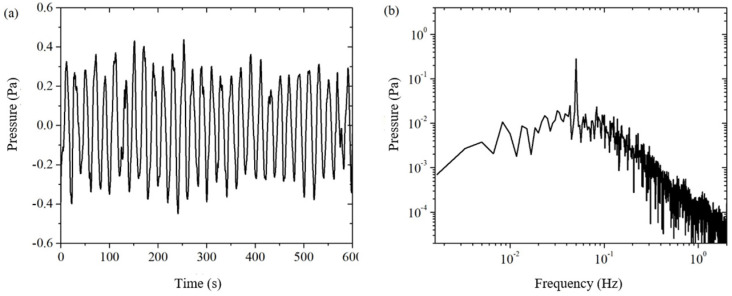
The electroosmotic pressure by the double pressure sensor method. (**a**) time domain; (**b**) frequency domain.

In order to further suppress the residual background noise, this paper uses spline fitting interpolation to do further data processing on the electroosmotic pressure signal $\Delta P_{E\_DPTM}^{\prime }$. The electroosmotic pressure signal $\Delta P_{E\_DPTM}^{\prime }$ after spline fitting interpolation is shown in Figure 8. Comparing the time-domain signals before and after the spline fitting interpolation in Figure 7a and Figure 8a, we can see that the slow drift of the signal is completely eliminated; comparing the frequency-domain signals in Figure 7b and Figure 8b, we can clearly see that the noise with a frequency lower than the excitation frequency is well suppressed. The spline-fit interpolation method not only significantly improves the time-domain signal by eliminating the drift, but also helps to reduce the noise of the same frequency at the excitation frequency. This is because the excitation frequency is an integer multiple (equal to the number of cycles of the collected electroosmotic pressure, in this case, 30 times) of the fundamental frequency of the drift (which is related to the total measurement duration, in this case, 1/600 Hz), and a harmonic component of the drift will be mixed into the excitation frequency to form a cochannel noise. By eliminating the drift, that harmonic component of the cochannel noise is eliminated. The principle of the spline fit interpolation method makes it more suitable for higher frequency bands, and in this paper, it is used in the frequency bands above 0.05 Hz. By reading the amplitude of the excitation frequency signal and the critical frequency noise in the frequency domain plot, it can be obtained $\Delta P_{E\_DPTM}^{\prime }=0.285\pm 0.026\;Pa$. The environment disturbances such as temperature are well suppressed through the above method.

### 3.5. Comparison of Single/Dual Pressure Sensor Methods

In the traditional single pressure sensor method, a pressure sensor is used to directly obtain the time-domain signal of the electroosmotic pressure, and then the electroosmotic pressure is extracted by lock-in amplification (LIA), which is recorded as Single + LIA method [19]. As can be seen from Equation (15), the electroosmotic pressure obtained by the single pressure sensor method is the measured value of the pressure sensor. Therefore, the solid line in Figure 5a is the electroosmotic pressure time domain signal of the Single method. In this paper, the Double Pressure Sensor method, using two pressure sensors, obtains the time-domain signal of electroosmotic pressure through the weighted difference of the double pressure sensors as well as the spline fitting interpolation and then extracts the electroosmotic pressure through the Fast Fourier Transform (FFT), which is noted as the Double + FFT method. From the above, the time-domain signal of electroosmotic pressure of the Double method is shown in Figure 8a.

**Figure 9:**
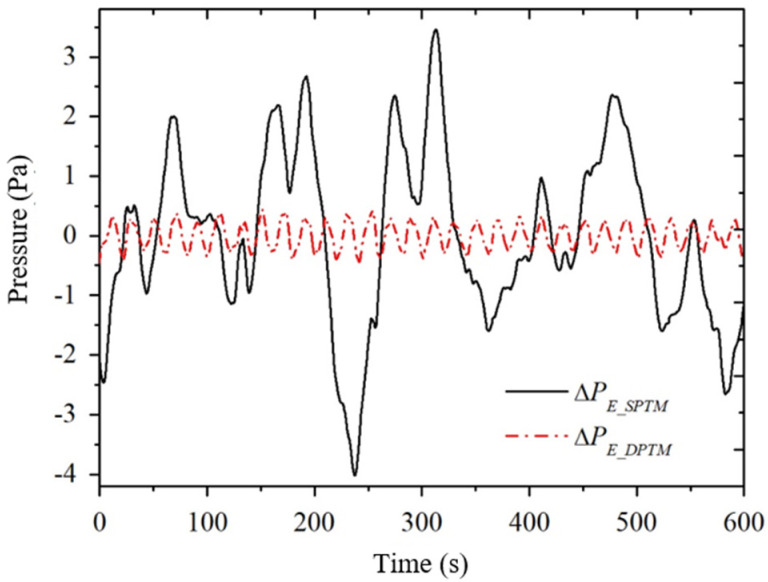
Comparison between single method (solid line) and double method (dashed line).

In order to compare the electroosmotic pressure time-domain signals of the Single and Double methods, we combine the time-domain signals of the two methods in Figure 9. The solid line in the figure is the time-domain signal of the Single method and the dashed line is the time-domain signal of the Double method. Comparing the two time-domain signals, it can be seen that the signal-to-noise ratio of the Double method is much higher than that of the Single method. LIA and FFT are two different data extraction methods, and their extracted data are formally different, so it is not intuitive to directly compare the Single + LIA method and the Double + FFT method. Therefore, this paper compares the Single method and Double method in terms of the FFT method and LIA method, respectively.

Let’s first compare the Single + FFT method with the Double + FFT method of this paper in terms of the FFT method, as shown in Figure 5 and Figure 8. The solid line in Figure 5a is the electroosmotic pressure time-domain signal of the Single method, then the value of the solid line in Figure 5b at the excitation frequency of 0.05 Hz is the amplitude of the electroosmotic pressure $\Delta P_{E\_SPTM}$ of the Single + FFT method. In order to evaluate the signal-to-noise ratio of the electroosmotic pressure signal, the maximum amplitude of the noise at several frequency points near the excitation frequency is selected as an estimate of the actual noise amplitude at the excitation frequency, which is obtained $\Delta P_{E\_SPTM}=0.20\pm 0.09\;\mathrm{Pa}$. Figure 8a is the time-domain signal of electroosmotic pressure measured by the Double method, and then the value of Figure 8b at the excitation frequency is the amplitude of electroosmotic pressure by the Double + FFT method. The electroosmotic pressure signal and its error estimation $\Delta P_{E\_DPTM}=0.285\pm 0.026\;\mathrm{Pa}$ of the Double + FFT method can be obtained by reading the frequency domain Figure 4, Figure 5, Figure 6, Figure 7 and Figure 8b.

Obviously, the signal-to-noise ratio of electroosmotic pressure obtained by the Double + FFT method in this paper is higher than that of the Single + FFT method.

**Figure 10:**
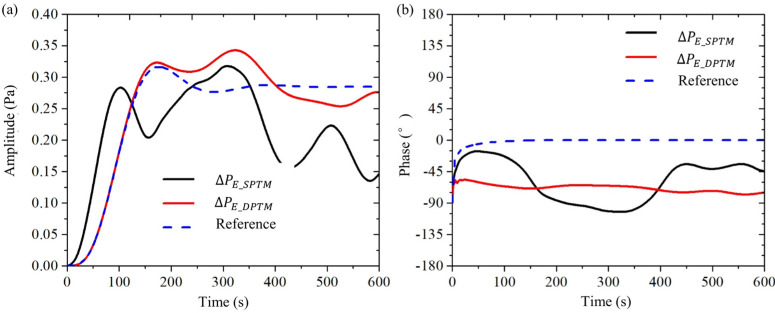
Comparison between single + LIA method (black solid line) and double + LIA method (red solid line) in the electroosmotic experiment. The dashed line is a reference signal. (**a**) pressure amplitude; (**b**) pressure phase.

Then, we compare the Double + LIA method with the traditional Single + LIA method from the aspect of the LIA method, as shown in Figure 10. In the figure, the black solid line is the output data of the traditional Single + LIA method, whose input data comes from the time-domain signals of the Single method; the red solid line is the output data of the Double + LIA method, whose input data comes from the time-domain signals of the Double method; and the dashed line is the output data of the reference group, whose input data is a purely sinusoidal signal, which is used for interpreting the meanings of the LIA output curves. The LIA needs to be parameterized for its low-pass filter. The low-pass filter for the example in the figure is a 4th-order inverse Chebyshev filter with a cutoff frequency of 0.03 Hz.

Figure 10b shows the three-phase curves of the lock-in amplifier LIA output. (1) The output data from the reference group (dashed line) converges quickly to 0° and then remains constant. This indicates that the lock-in amplifier detects a sinusoidal signal with a phase value of 0° and no noise. (2) The output data (solid red line) of the Double + LIA method oscillates slightly between −60° and −75°. This indicates that the electroosmotic pressure signal obtained from the Double method is detected by the lock-in amplifier with a phase value between −60° and −75° and little noise. (3) The output data (black solid line) of the Single + LIA method oscillates between −15° and 105°. This indicates that the electroosmotic pressure signal obtained by the Single method is detected by the lock-in amplifier, but it is noisy. Comparing the red solid line with the black solid line, it can be seen that the signal-to-noise ratio of the Double + LIA method is higher than that of the Single + LIA method.

Figure 10a shows three amplitude curves of the lock-in amplifier LIA output. (1) The output data of the reference group (dashed line), first increases rapidly from zero, then oscillates, and finally stabilizes at 0.285 Pa. This indicates that the sinusoidal input signal is detected by the lock-in amplifier with a magnitude of 0.285 Pa and no noise. (2) The output data of the Double + LIA method (red solid line), first increases rapidly from zero, then oscillates between 0.25 and 0.34 Pa, and finally settles at 0.275 Pa. This indicates that the electroosmotic pressure signal has an amplitude of about 0.275 Pa with little noise. (3) The output data of the Single + LIA method (black solid line), first increases rapidly from zero, then oscillates between 0.13 and 0.32 Pa, and finally fluctuates approximately sinusoidally between 0.13 Pa and 0.225 Pa. This indicates that the electroosmotic pressure signal has an amplitude of about (0.13 + 0.225)/2 Pa ≈ 0.18 Pa and has a large amount of noise. Comparing the red solid line with the black solid line, it can be seen that the signal-to-noise ratio of the Double + LIA method is better than that of the Single + LIA method.

From both phase and amplitude, it can be seen that the signal-to-noise ratio of electroosmotic pressure obtained by the Double + LIA method is higher than that of the conventional Single + LIA method.

In addition, comparing the results of the Double + FFT and Double + LIA methods, it can be found that the amplitude of the extracted signals is about the same for the data extracted using the FFT method and the data extracted using the LIA method, but the amplitude and the error estimation can be obtained automatically using the FFT method, while the amplitude needs to be obtained manually and it is difficult to obtain the quantitative error estimation using the LIA method. The same is true when comparing the results of the Single + FFT method and the Single + LIA method.

## 4. Experimental Results and Analysis

### 4.1. The Relationship between Electroosmotic Pressure Coefficient and Excitation Frequency

**Figure 11:**
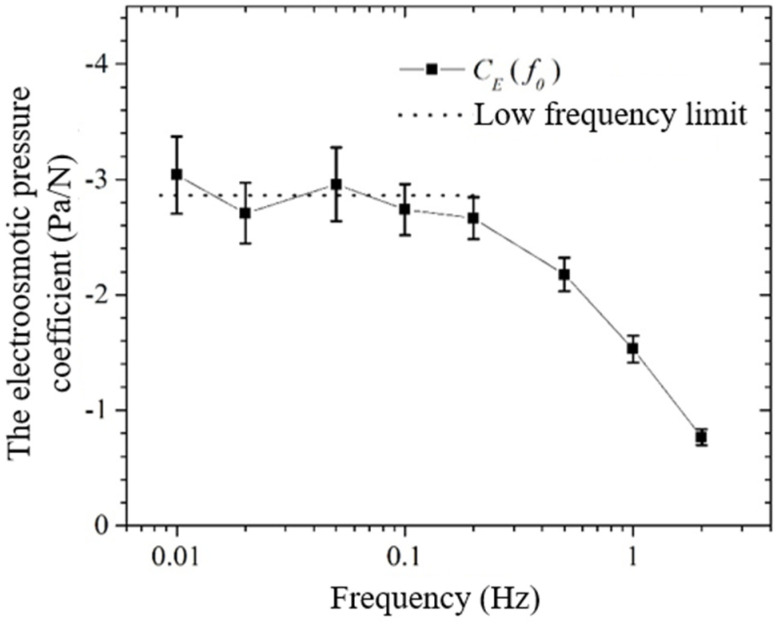
The electroosmotic pressure coefficient with the varying frequency.

In this paper, the electroosmotic pressure coefficients of 20 rock samples were measured at eight different mineralization, i.e., distilled water, 0.01 mol/L, 0.02 mol/L, 0.05 mol/L, 0.1 mol/L, 0.2 mol/L, 0.4 mol/L, and 0.6 mol/L NaCl saturated solutions, as shown in Table 1. Figure 11 shows a set of plots of electroosmotic pressure coefficients $C_{E}(f_{0})$ versus excitation frequency $f_{0}$ from 0.01 Hz to 2 Hz, in which the rock sample is numbered as S12 and the mineralization is 0.4 mol/L. In the figure, the absolute value of $C_{E}(f_{0})$ in the frequency domain from 0.2 Hz to 2 Hz becomes larger with the decrease in the frequency, which suggests that the system has not reached the equilibrium state at this time; in the frequency domain from 0.01 Hz to 0.1 Hz, the absolute value of $C_{E}(f_{0})$ remains almost constant, which indicates that the system has reached equilibrium at this point. A similar phenomenon exists in the electroosmotic experiments of Wang Jun et al. [9,20] For the case in Figure 11, the upper-frequency limit for the system to reach equilibrium is about 0.1 to 0.2 Hz. A large number of experimental results show that the upper-frequency limit for the system to reach equilibrium is affected by the properties of the sample itself. For the samples used in this paper, the lowest upper-frequency limit for the equilibrium state of the system is about 0.01 Hz. In this paper, the lowest frequency limit that can be measured is 0.001 Hz, so it can be guaranteed that the system can reach the equilibrium state. In order to reduce the experimental error, this paper on all the systems to reach the equilibrium state of the electroosmotic pressure coefficient of the measured value to do the average, and the average value as the electroosmotic pressure coefficient $C_{E0}$ of the rock sample.

### 4.2. The Relationship between Electroosmotic Pressure Coefficient and Mineralization Degree

**Figure 12:**
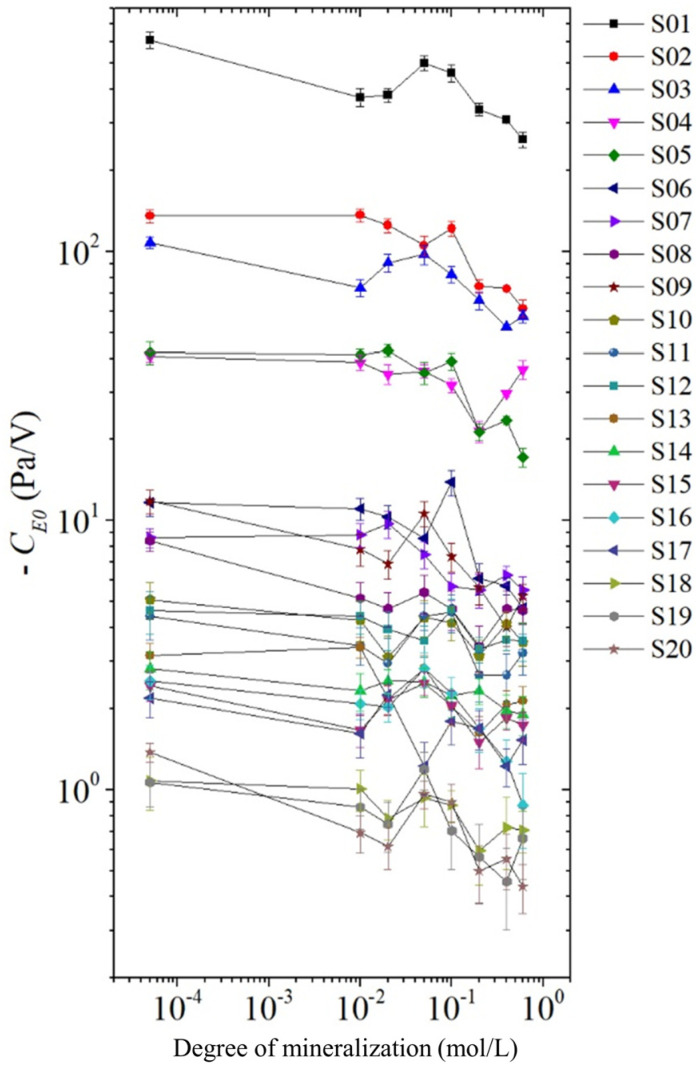
The electroosmotic pressure coefficient with the varying mineralization.

The absolute value of the electroosmotic pressure coefficient decreases slightly with increasing solution mineralization, as shown in Figure 12. This is because for the same rock sample, the permeability, porosity, cross-sectional area and length of the rock sample are constant, and the electroosmotic pressure coefficient $C_{E0}$ is mainly affected by electroosmotic seepage $v_{E}$. For the same rock sample, the amount of net remaining charge does not change, and the magnitude of electroosmotic seepage $v_{E}$ depends on the velocity of the net remaining charge. When the excitation voltage is certain, the strength of the electric field in the rock sample is constant, and the velocity of the net remaining charge depends on its distance from the solid-liquid interface. The greater the mineralization, the closer the net remaining charge is to the solid-liquid interface, the greater the resistance it receives, and consequently the slower it is, finally leading to a smaller absolute value of the electroosmotic pressure coefficient $C_{E0}$.

### 4.3. The Relationship between Electroosmotic Pressure Coefficient and Permeability and Porosity

**Figure 13:**
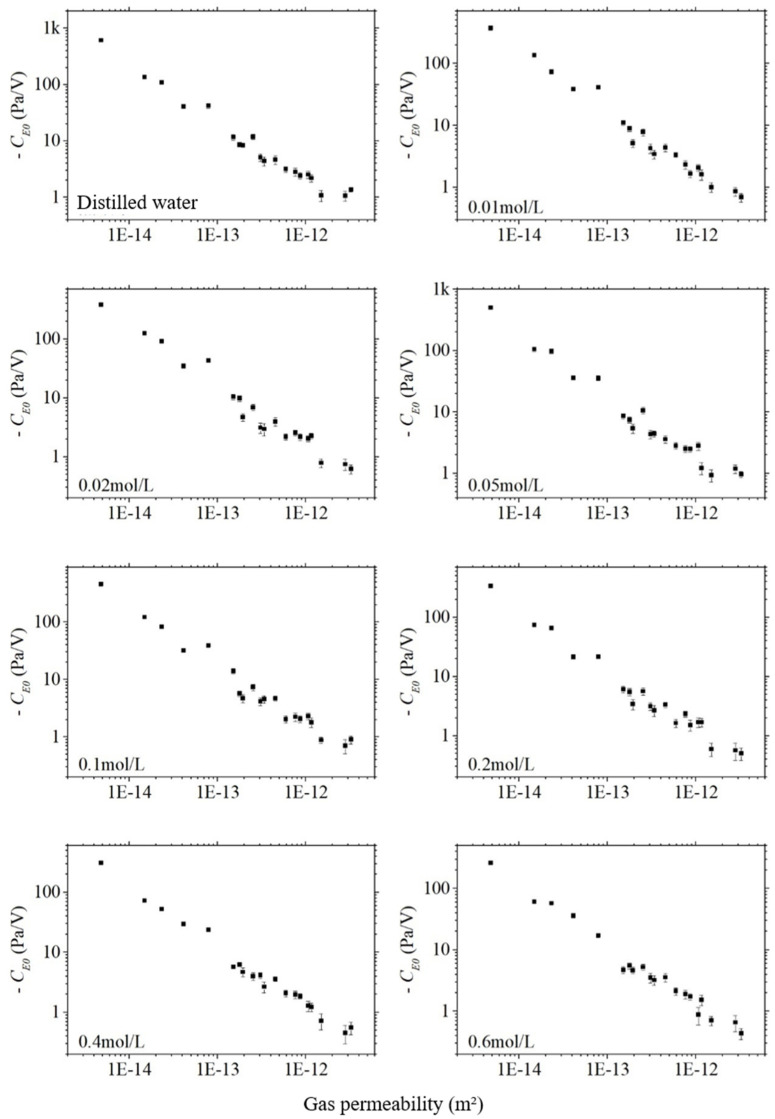
The electroosmotic pressure coefficient with the varying gas permeability of sandstone samples in different salinities.

For all mineralization, the absolute values of the electroosmotic pressure coefficients $C_{E0}$ decrease approximately linearly in logarithmic coordinates with increasing gas permeability $\kappa_{gas}$, as shown in Figure 13. The absolute values of the electroosmotic pressure coefficient and the gas permeability of the sandstone and artifactual samples were first taken in logarithmic coordinates, and then correlation analyses were completed, and the correlation coefficients obtained ranged from −0.98 to −0.99, i.e., the electroosmotic pressure coefficient was strongly correlated with the permeability. This is because the electroosmotic pressure obeys Darcy’s law, which is inversely proportional to the permeability. Since the permeability varies greatly from rock sample to rock sample, exceeding the other factors by several orders of magnitude, the effect of the other factors can be masked. Therefore, the electroosmotic effect is expected to be a method to invert the permeability outside the well. Hu et al. [9] numerically simulated the electro-acoustic logging wavefield in a homogeneous formation based on the electroosmotic effect. 

**Figure 14:**
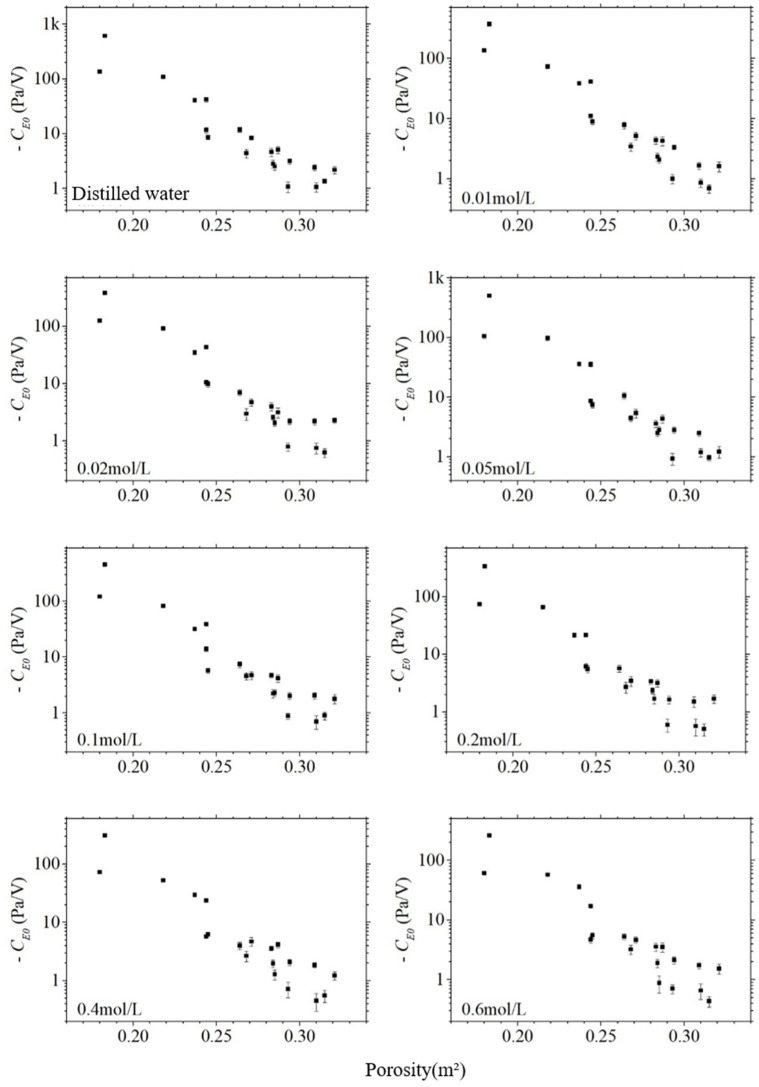
The electroosmotic pressure coefficient with the varying porosity of sandstone samples in different salinities.

The absolute value of the electroosmotic pressure coefficient $C_{E0}$ also decreases significantly with the increase in porosity ϕ, as shown in Figure 14, which is consistent with the trend of its change with permeability. The logarithm of the absolute value of the electroosmotic pressure coefficient is correlated with the porosity, and the correlation coefficients of the sandstone samples are from −0.93 to −0.96, which means that the electroosmotic pressure coefficient is strongly correlated with the porosity. It can be seen that the electroosmotic effect is also expected to be a method to invert the porosity outside the well. Comparing Figure 13 and Figure 14, it can be found that the data on porosity are relatively more scattered, which indicates that the correlation between the electroosmotic effect and permeability is a little bit closer.

## 5. Conclusions

Electroosmotic experiments require the measurement of excitation voltage and Electroosmotic pressure, which is a weak signal and is the key to the experiment. The conclusions obtained in this paper are summarized as follows:

(1) The dual pressure sensor method is proposed for suppressing the background noise of electroosmotic pressure. The dual pressure sensor method involves the use of two low-range pressure sensors of the same model, one for measuring the electroosmotic pressure signal and the other for measuring the background noise. It was found that there was a good correlation between the background noise of the two pressure transducers, but they were not numerically equal, so it was necessary to measure the conversion factor of both first. Afterward, the measured electroosmotic pressure signal was subtracted from the corrected background noise to obtain a higher signal-to-noise ratio electroosmotic pressure.

(2) Electroosmotic experiments of 20 rock samples saturated with distilled water to 0.6 mol/L NaCl solution were measured, and the relationship between the Electroosmotic pressure coefficient and rock parameters such as permeability was analyzed and discussed. The experiments show that the absolute value of the electroosmotic pressure coefficient is negatively correlated with permeability as well as porosity. For the mineralization of the actual formation, the electroosmotic pressure coefficient is sensitive to the permeability.

## Figures and Tables

**Table 1:** Parameters of sandstone samples.

Number	Type	Density g/cm^3^	Porosity %	Permeability ×10^−15^ m^2^
S01	Grayish-white fine sandstone	2.62	18.3	4.8
S02	Grayish-white fine sandstone	2.68	18.0	14.9
S03	Grayish-white fine sandstone	2.67	21.8	23.3
S04	Gray fine sandstone	2.67	23.7	41.2
S05	Grayish-white medium sandstone	2.64	24.4	78.7
S06	Grayish-white fine sandstone	2.67	24.4	151
S07	Grayish-white fine sandstone	2.67	24.5	178
S08	Grayish-white medium sandstone	2.61	27.1	194
S09	Grayish-white medium sandstone	2.63	26.4	253
S10	Grayish-white medium sandstone	2.63	28.7	306
S11	Grayish-white medium sandstone	2.69	26.8	337
S12	Grayish-white medium sandstone	2.65	28.3	453
S13	Grayish-white coarse sandstone	2.65	29.4	594
S14	Grayish-white coarse sandstone	2.62	28.4	762
S15	Gray Coarse Sandstone	2.63	30.9	862
S16	Gray Coarse Sandstone	2.61	28.5	1066
S17	Grayish-white coarse sandstone	2.61	32.1	1152
S18	Grayish-white medium sandstone	2.65	29.3	1491
S19	Grayish brown coarse sandstone	2.61	31.0	2785
S20	Grayish brown coarse sandstone	2.62	31.5	3241

## Data Availability

The datasets used and/or analyzed during the current study are available from the authors upon reasonable request.
